# Lymphovascular Invasion Is a Predictor of Clinical Outcomes in Bladder Cancer Patients Treated with Radical Cystectomy

**DOI:** 10.3390/jcm14145120

**Published:** 2025-07-18

**Authors:** Daniel-Vasile Dulf, Anamaria Larisa Burnar, Patricia-Lorena Dulf, Doina-Ramona Matei, Raluca Maria Hendea, Iulia Andraș, Miruna Grecea, Cătălina Bungărdean, Antonio De Leo, Tudor-Eliade Ciuleanu, Nicolae Crișan, Camelia Alexandra Coada

**Affiliations:** 1 10th Department—Oncology, Iuliu Haţieganu University of Medicine and Pharmacy, 400015 Cluj-Napoca, Romania; 2 Clinical Municipal Hospital Cluj-Napoca, 400139 Cluj-Napoca, Romania; 3 “Prof. Dr. Ion Chiricuță” Institute of Oncology, 40015 Cluj-Napoca, Romania; anamaria.lari.burnar@elearn.umfcluj.ro (A.L.B.);; 4 “Prof. Dr. Nicolae Stăncioiu” Heart Institute, 400370 Cluj-Napoca, Romania; 5 Department of Pathology, Iuliu Haţieganu University of Medicine and Pharmacy, 400012 Cluj-Napoca, Romania; 6 Department of Urology, Iuliu Haţieganu University of Medicine and Pharmacy, 400012 Cluj-Napoca, Romania; drnicolaecrisan@gmail.com; 7 Solid Tumor Molecular Pathology Laboratory, IRCCS Azienda Ospedaliero-Universitaria di Bologna, 40138 Bologna, Italy; 8 Department of Medical and Surgical Sciences (DIMEC), University of Bologna, 40138 Bologna, Italy; 9 Department of Morpho-functional sciences, Iuliu Hațieganu University of Medicine and Pharmacy, 400347 Cluj-Napoca, Romania; coada_camelia_alexandra@elearn.umfcluj.ro

**Keywords:** urothelial carcinoma, lymphovascular invasion, survival, prognosis

## Abstract

**Background/Objectives:** Lymphovascular invasion (LVI) has been consistently linked to poor outcomes in patients with bladder cancer (BC), yet its independent prognostic value, especially after adjusting for established pathological features, remains debated. This study aimed to evaluate the prognostic value of LVI in the context of other pathological features of patients undergoing radical cystectomy. **Methods**: We conducted a retrospective cohort study including 200 patients treated at the Municipal Clinical Hospital in Cluj-Napoca, Romania. Associations between LVI and overall survival (OS) were assessed using univariable and multivariable Cox proportional hazards models, with Kaplan–Meier curves used for visualizing survival distributions. **Results:** In univariable analysis, increasing age, presence of LVI, advanced pathological tumor stage (pT ≥ 2), and nodal involvement (pN ≥ 1) were significantly associated with worse OS. LVI was a strong predictor of poor survival (HR 3.13; 95% CI: 2.09; 4.69; *p* < 0.001). However, in multivariable analysis, only tumor stage (HR 4.85; 95% CI: 2.19; 10.77; *p* < 0.001) and nodal involvement (HR 1.87; 95% CI: 1.13; 3.09; *p* = 0.015) remained independently associated with OS. In patients with incomplete nodal staging (Nx), LVI was significantly associated with OS (*p* = 0.028). **Conclusions:** Our findings reinforce the prognostic relevance of LVI in bladder cancer and support its role as a marker of aggressive tumor biology, highlighting its value in clinical risk assessment, especially in patients with incomplete nodal staging. Routine reporting of LVI in pathology and consideration in treatment planning are warranted.

## 1. Introduction

Bladder cancer is among the most common malignancies worldwide, with muscle-invasive bladder cancer (MIBC) representing a particularly aggressive form of the disease [1,2]. Radical cystectomy remains the gold-standard treatment for MIBC and select high-risk non-muscle-invasive bladder cancers; however, oncologic outcomes vary widely, even among patients with similar clinical and pathological stages. This heterogeneity underscores the need for additional prognostic markers that can more accurately predict patient outcomes and guide individualized treatment strategies [3].

Lymphovascular invasion (LVI), defined as the presence of tumor cells within lymphatic and/or vascular channels, has emerged as a critical histopathological feature indicative of tumor aggressiveness across a spectrum of solid malignancies [4,5,6], including bladder cancer [7]. Its detection reflects the biological capacity of tumor cells to breach the endothelial barrier, facilitating dissemination through lymphatic and blood vessels, which is a pivotal step in metastatic progression [8]. In bladder cancer, numerous studies have demonstrated a significant association between LVI and adverse pathological characteristics such as higher tumor stage, lymph node metastasis [9], and positive surgical margins [10], underscoring its role in tumor progression and spread [11]. For instance, LVI has been reported in approximately 25–50% of radical cystectomy specimens, correlating with increased rates of disease recurrence, cancer-specific mortality, and poorer progression-free (PFS) and overall survival (OS) outcomes [12,13,14].

Despite accumulating evidence, the prognostic significance of LVI in bladder cancer patients undergoing radical cystectomy remains incompletely defined, with ongoing debate regarding its role as an independent predictor of survival, especially after adjusting for pT and pN stages. Meta-analyses encompassing large patient cohorts have confirmed that LVI is strongly associated with worse oncologic outcomes, including recurrence and mortality, even in lymph node-negative disease [15], highlighting its potential utility as a biomarker to guide postoperative management [13]. Furthermore, LVI’s prognostic impact appears comparable to that of lymph node involvement, suggesting that its presence could refine risk stratification beyond conventional staging parameters [13]. Given its biological and clinical implications, integrating LVI status into routine pathological evaluation and treatment algorithms could enhance personalized therapeutic strategies, particularly in identifying high-risk patients who may benefit from closer surveillance or adjuvant therapies [12].

The aim of this study was to evaluate the prevalence and prognostic significance of LVI in a cohort of bladder cancer patients treated with radical cystectomy at a high-volume tertiary center in Romania. Specifically, we investigated whether LVI serves as an independent predictor of overall survival after adjusting for established clinicopathologic factors.

## 2. Methods

### 2.1. Study Design and Patient Selection

This was a unicentric, retrospective, analytical study conducted using data from patients diagnosed with bladder cancer. The study was approved by the institutional review board (code 4/2025).

Patients were included in the study if they met the following criteria: (i) underwent radical cystectomy with or without pelvic lymph node dissection (PLND) at our institute; (ii) histologically confirmed diagnosis of bladder cancer; (iii) availability of complete clinical records; (iv) availability of survival data; (v) availability of a detailed pathological report from the cystectomy specimen; (vi) assessment of LVI status documented in the pathological report.

Exclusion criteria were (i) history of prior malignancy within 5 years, with the exception of non-melanoma skin cancer or carcinoma in situ of the cervix; (ii) prior systemic chemotherapy or immunotherapy for other malignancies; (iii) previous pelvic radiotherapy; (iv) incomplete or missing data for key variables required for the analysis (e.g., LVI status, pT stage, pN stage, survival); (v) evidence of distant metastatic disease (M1) at diagnosis before radical cystectomy. The patient selection process is detailed in the STROBE flow diagram (Supplementary Figure S1).

### 2.2. Data Collection

Clinical and pathological data were retrospectively reviewed from the medical records of patients who underwent radical cystectomy for histologically confirmed bladder cancer at the Clinical Municipal Hospital Cluj-Napoca, Cluj-Napoca, Romania, between January 2012 and November 2024. The timeframe was estimated to ensure at least 10 events per each variable considered for the survival analysis, with an attrition rate of 10%. Pathology reports were reviewed to extract tumor histological type, stage, grade, lymph node status, surgical margin status, and presence of LVI. Survival outcomes were obtained from the institutional communal records.

LVI was defined as the presence of tumor emboli within lymphatic and/or blood vessels. LVI status was assessed histologically on hematoxylin and eosin (H&E)-stained pathology slides. Data on LVI status was determined based on data extracted from the final, original pathology reports generated at the time of diagnosis. For this retrospective study, a central, systematic re-review of histopathology slides was not performed. Importantly, all original pathological assessments at our institution were performed by dedicated genitourinary (GU) pathologists, ensuring a high level of expertise in the primary evaluation.

For the oncological assessment, bladder cancer staging was performed in accordance with the American Joint Committee on Cancer (AJCC)/Union for International Cancer Control (UICC) TNM classification system (7th edition until 2017, and the 8th edition thereafter) [16,17]. Positive lymph nodes were defined by either histopathologic confirmation post-surgery or radiologic suspicion on computed tomography (CT) imaging prior to or after surgery. The assessment for distant metastases (M1 status) was conducted using CT of the thorax, abdomen, and pelvis. Histological classification of the tumors was based on the World Health Organization (WHO) 2004 criteria [18], with a progressive adoption of the updated WHO 2016 classification [19] during the study period, which provided refined tumor grading and recognized variant histologies.

### 2.3. Peri-Operative Systemic Therapy

Decisions regarding the administration of peri-operative chemotherapy (both neoadjuvant and adjuvant) were made on a case-by-case basis by the multidisciplinary tumor board, in accordance with the prevailing clinical practice guidelines at the time of treatment. Key factors influencing this decision included clinical and pathological tumor stage, the patient’s performance status, renal function (for cisplatin eligibility), and shared decision-making with the patient. This approach reflects real-world clinical practice over the extended study period but was not based on a single, rigid investigational protocol.

### 2.4. Statistical Analysis

Statistical analyses were performed in R version 4.4.3 (28 February 2025 ucrt)—“Trophy Case” [20]. Continuous variables were summarized using medians and quartiles 1 and 3. Kolmogorov–Smirnov was used to test for normality. Differences between groups were assessed using *t*-tests or Mann–Whitney tests as appropriate. Categorical variables were summarized as absolute frequency and percent. Categorical variables were described using frequencies and percentages. OS was defined as the time between patient diagnosis and time of death or last known alive. Death of any cause was considered an event, while patients still alive at the time of the analysis were censored. PFS was defined as the time between patient diagnosis and disease recurrence/progression (representing the event). Patients without the event at the last available follow-up were censored. OS and PFS were presented using Kaplan–Meier curves, and significance was tested using the generalized Wilcoxon test. Median follow-up time was calculated using the reverse Kaplan–Meier method. Univariable Cox regression analyses were conducted to identify factors associated with OS. Proportional hazards assumptions were tested for all variables (Supplementary Table S1). Variables that were significant in univariable analysis and deemed clinically relevant were included in the multivariable Cox model. Hazard ratios (HRs) and 95% confidence intervals (CIs) were reported for each analysis. A two-sided *p*-value < 0.05 was set for statistical significance.

## 3. Results

### 3.1. Patient Characteristics

A total of 213 patients underwent radical cystectomy for bladder cancer at the Clinical Municipal Hospital Cluj-Napoca between January 2012 and November 2024. From this initial cohort, a total of 13 unique patients were excluded. The reasons for exclusion included cystectomy performed for non-oncological indications such as bladder diverticula, benign tumors, and neurogenic bladder. After applying these criteria, a final cohort of 200 patients was included in the present analysis (Supplementary Figure S1), out of which 122 (61%) were LVI-negative and 78 (39%) were LVI-positive patients.

Median follow-up time was 66.73 (IQR: 35.73; 91.00) months. There were no significant differences between the two groups in terms of age, sex, smoking status, residential background, comorbidities (including hypertension and diabetes), or rates of pre- and postoperative transfusions (all *p* > 0.05). The majority (58%) of patients were treated with cystectomy without systemic chemotherapy. Notably, patients with LVI were less likely to receive neoadjuvant chemotherapy (14.7% vs. 33.6%, *p* = 0.001), but more likely to receive adjuvant chemotherapy (24% vs. 9%, *p* = 0.001) (Table 1).

Pathologically, LVI-positive tumors were more frequently high-grade (G3 or G4; 91.8% vs. 67%, *p* < 0.001) and exhibited a more advanced pathological stage. None of the LVI-positive tumors were ≤pT1, whereas 48.8% of LVI-negative tumors were ≤pT1 (*p* < 0.001). Similarly, nodal metastases were significantly more common in the LVI-positive group (49.2% vs. 6.5%; *p* < 0.001). No significant differences were found in histological subtype or perineural invasion between the groups (Table 1).

### 3.2. Factors Associated with the Prognosis of Bladder Cancer Patients

**Figure 1:**
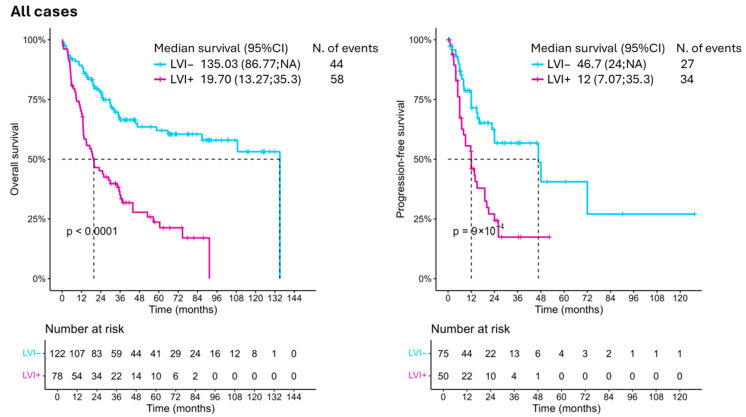
Kaplan–Meier curves showing the overall survival and progression-free survival of BC patients included in this study, divided based on their LVI status. LVI: lymphovascular invasion.

Univariable COX regression analysis identified several variables significantly associated with survival. Increasing age was associated with a higher hazard of the event (HR 1.03, 95% CI: 1.01; 1.06, *p* = 0.009). LVI, higher pathological tumor stage (pT ≥ 2), and nodal involvement (pN ≥ 1) were all strongly associated with worse outcomes, with HRs of 3.13 (95% CI: 2.09; 4.69, *p* < 0.001), 6.58 (95% CI: 3.31; 13.1, *p* < 0.001), and 3.42 (95% CI: 2.16; 5.4, *p* < 0.001), respectively. Tumor grade showed a trend toward significance (HR 1.58, 95% CI: 0.93; 2.67, *p* = 0.088) (Figure 1; Table 2).

In the multivariable Cox regression model, after adjusting for confounders, only pathological tumor stage (pT ≥ 2, HR 4.85, 95% CI: 2.19; 10.77, *p* < 0.001) and nodal involvement (pN ≥ 1, HR 1.87, 95% CI: 1.13; 3.09, *p* = 0.015) remained independently associated with OS. LVI and other variables did not reach statistical significance in the multivariable model (Table 2). When analyzing the PFS, perioperative chemotherapy was a protective factor (HR 0.5, 95%CI: 0.29; 0.87, *p* = 0.015) while higher T stages were associated with a higher risk of recurrence (HR 5.52, 95%CI: 2.04; 14.93, *p* = 0.001) (Figure 1; Table 2).

### 3.3. Factors Associated with the Prognosis of Bladder Cancer Patients Treated with Cystectomy Without Systemic Chemotherapy

To further refine our analysis, we focused on patients with bladder cancer treated with radical cystectomy in the absence of systemic chemotherapy.

**Figure 2:**
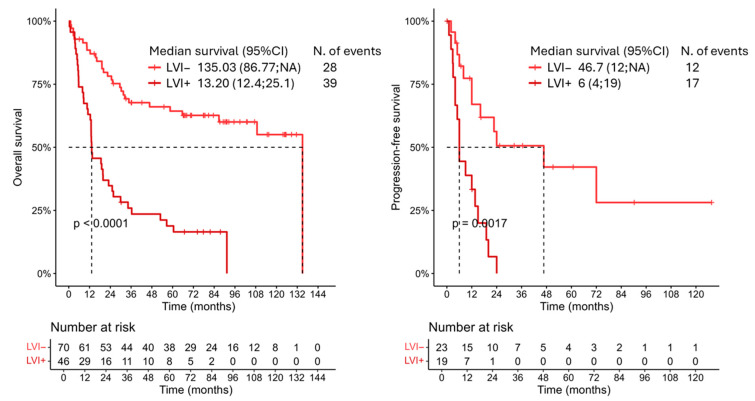
Kaplan–Meier curves showing the overall survival and progression-free survival of bladder cancer patients treated with cystectomy only, divided based on their LVI status. LVI: lymphovascular invasion.

In the univariable analysis, increasing age was significantly associated with worse survival (HR 1.03; 95% CI: 1.00; 1.06; *p* = 0.023). LVI, higher pathological tumor stage (pT ≥ 2), and nodal involvement (pN ≥ 1) were all strongly associated with poorer outcomes, with hazard ratios of 4.00 (95% CI: 2.41; 6.66; *p* < 0.001), 6.70 (95% CI: 3.04; 14.73; *p* < 0.001), and 4.17 (95% CI: 2.35; 7.39; *p* < 0.001), respectively. Other variables, including sex, smoking status, comorbidities, post-surgical complications, and tumor grade, did not reach statistical significance (Figure 2; Table 3).

In the multivariable model, after adjusting for potential confounders, only pathological tumor stage (pT ≥ 2: HR 4.81; 95% CI: 1.85; 12.49; *p* = 0.001) and nodal involvement (pN ≥ 1: HR 1.92; 95% CI: 1.02; 3.62; *p* = 0.015) remained independently associated with overall survival. The association between LVI and OS did not remain statistically significant in the adjusted model (HR 1.66; 95% CI: 0.84; 3.30; *p* = 0.143). Age showed a non-significant trend (HR 1.02; 95% CI: 0.99; 1.06; *p* = 0.160), and all other variables remained non-significant (Table 3). Presentation of the survival curves of all patients divided by their treatment type is reported in Supplementary Figure S2. Similar results were obtained when analyzing the PFS (Figure 2; Table 3).

### 3.4. Factors Associated with the Prognosis of Bladder Cancer Patients with Negative Lymph Nodes (N0)

**Figure 3:**
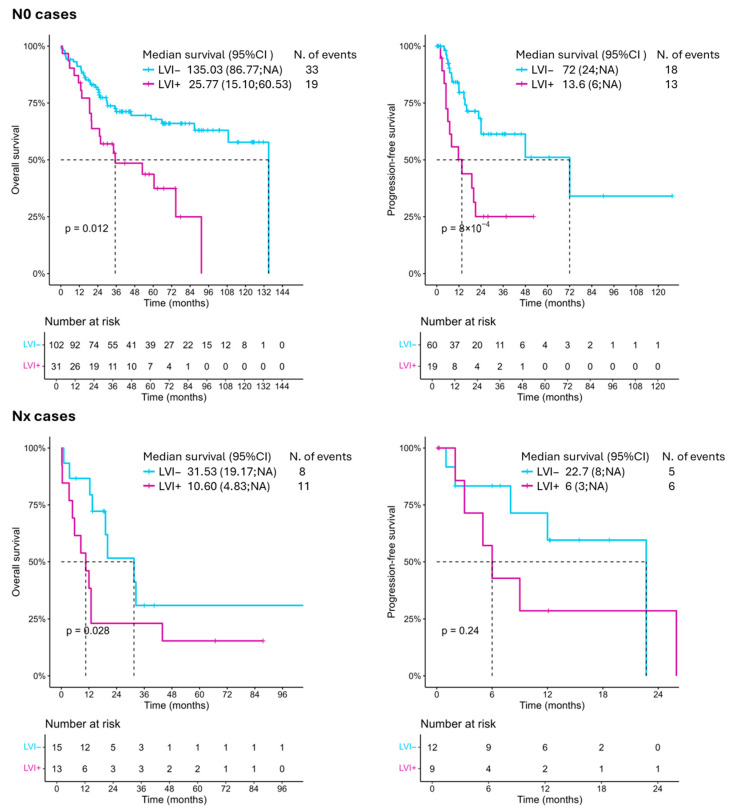
Kaplan–Meier curves showing the overall survival and progression-free survival of patients included in this study, divided based on their LVI status and lymph node status (N0 and Nx). LVI: lymphovascular invasion.

Analysis of bladder cancer patients with negative lymph nodes revealed that increasing age (HR 1.03; 95% CI: 1.00; 1.06; *p* = 0.036), LVI (HR 2.71; 95% CI: 1.68; 4.37; *p* < 0.001), higher pathological tumor stage (pT ≥ 2) (HR 5.44; 95% CI: 2.7; 11; *p* < 0.001) were associated with poor OS (Figure 3). In the multivariable model, LVI again lost its significant impact (Table 4). Analysis of patients with positive lymph nodes showed no significant results (Supplementary Figure S3). Similar results were seen for PFS (Figure 3; Table 4).

### 3.5. Factors Associated with the Prognosis of Bladder Cancer Patients with Unknown Lymph Node Status (Nx)

Next, we sought to analyze patients who had an indeterminate lymph node status (Nx) (*n* = 28) since in these cases, the most relevant driver for prognosis is unknown. Kaplan–Meier survival analysis showed a significantly reduced OS in patients with LVI-positive tumors (*p* = 0.028), highlighting LVI as a potential surrogate prognostic marker in the Nx population (Figure 3). In the univariable Cox analysis, LVI-positive status was associated with a trend toward poorer OS compared to LVI-negative patients (HR (95%CI) = 2.23 (0.88; 5.66); *p* = 0.091). Other variables, including tumor grade and tumor stage, were not statistically significant predictors of OS or PFS in the univariable model (Table 5).

## 4. Discussion

In this study, we investigated the prognostic significance of LVI alongside other clinicopathological factors in patients with bladder cancer undergoing radical cystectomy. Our univariable Cox regression analysis revealed that increasing age, presence of LVI, higher pathological tumor stage (pT ≥ 2), and nodal involvement (pN ≥ 1) were all significantly associated with worse PFS and OS. However, in multivariable analysis, only higher tumor stage and nodal involvement remained independently associated with OS, while the statistical significance of LVI was lost. This suggests that LVI’s impact on survival may be mediated through its association with more advanced disease, a pattern consistently reported in the literature, where LVI frequently coexists with high tumor burden and nodal metastasis, potentially diminishing its independent prognostic effect when controlling for these variables [13,21,22]. These results reinforce the well-established prognostic roles of tumor stage and nodal status, which continue to be key determinants of outcome in bladder cancer [23].

Our findings regarding LVI are strongly supported by some of the largest and most robust investigations in the field. For instance, Lotan et al. demonstrated in a cohort of 1167 patients that LVI was independently associated with OS, cancer-specific survival, and recurrence, particularly among node-negative patients [24]. Similarly, Bolenz et al., in a multicenter cohort of 1206 lymph node-negative patients, validated LVI as an independent predictor of oncological outcomes, underscoring its importance for risk stratification even in the absence of nodal metastases [15].

Meta-analytic data further strengthens the prognostic relevance of LVI. Mari et al. in a systematic review and meta-analysis involving over 6100 patients, reported that LVI in transurethral resection specimens was associated with disease progression and reduced survival, independent of other pathological features [13]. Likewise, Canter et al. showed in 712 patients that LVI in cystectomy specimens significantly predicted poor clinical prognosis [7]. Abufaraj et al., analyzing 2219 patients, found that LVI identified at the time of transurethral resection strongly predicted both recurrence and progression, further confirming its utility even at earlier disease stages [25].

More recently, Gallardo et al. demonstrated the value of integrating LVI into predictive models. In their external validation of the Cancer of the Bladder Risk Assessment (COBRA) score, which included 1159 patients, the incorporation of LVI significantly improved the accuracy of cancer-specific survival predictions following radical cystectomy [26]. These findings highlight the clinical utility of LVI as a complement to established prognostic indicators.

A notable aspect of our cohort is the relatively high proportion of patients with indeterminate nodal status (Nx), accounting for 14% of cases that reflect real-world practice, where complete lymph node dissection is not always feasible. Importantly, in the Nx subgroup, LVI retained its prognostic value, which is consistent with the aforementioned studies and meta-analyses suggesting that LVI can serve as a surrogate marker of tumor aggressiveness when nodal staging is incomplete or unavailable. We specifically chose to analyze this subgroup independently, given that the absence of definitive nodal information, one of the most powerful prognosticators, creates a clinical context in which markers like LVI become especially relevant for risk assessment and treatment planning.

Taken together, our findings add to the substantial body of evidence supporting LVI as a meaningful prognostic factor in bladder cancer. Although its independent significance may diminish in multivariable models that include detailed staging information, LVI remains a marker of biologically aggressive disease and should continue to be routinely reported in pathological assessments. Its inclusion in prognostic models such as COBRA may further enhance individualized patient counseling and therapeutic decision-making, particularly in cases with incomplete nodal evaluation.

## 5. Clinical Implications and Future Directions

Our findings demonstrate that LVI is significantly associated with poorer overall survival in patients undergoing radical cystectomy without adjuvant chemotherapy in a cohort of patients treated at a high-volume center in Romania. This underscores the need to incorporate LVI status into postoperative risk stratification algorithms. Rather than being discharged with routine surveillance alone, patients with LVI should be evaluated for adjuvant treatment options, including chemotherapy and/or immunotherapy, in order to address their elevated risk of recurrence and mortality. Moreover, the strong association between LVI and lymph node positivity has important clinical implications for cases where PLND is omitted or insufficiently assessed. In patients with an unknown nodal status (Nx), the presence of LVI may serve as a surrogate indicator for the possible presence of occult disseminating disease. Therefore, LVI-positive patients, especially those with insufficient nodal evaluation, should be considered high-risk by default and evaluated for adjuvant systemic therapies.

Current clinical guidelines, including those from the EAU [27] and NCCN [28], acknowledge LVI as a potential adverse prognostic factor but do not yet incorporate it as a definitive indication for adjuvant therapy in node-negative patients. Incorporating LVI into formal risk models could help refine treatment algorithms and ensure that high-risk patients are not undertreated. These findings underscore the potential utility of LVI as a prognostic indicator in the absence of confirmed lymph node staging, warranting further evaluation in multivariable models and larger cohorts, with special focus on patients with no confirmed LN staging. Moreover, since patients with lymph node-positive disease derive substantial benefit from NAC [29,30], the presence of LVI in the initial TUR specimen could serve as an early surrogate marker to guide clinical decision-making in non-metastatic patients. Specifically, LVI may help identify those who are more likely to benefit from NAC prior to radical cystectomy, rather than proceeding directly to surgery.

Furthermore, the limitations of histopathological assessment of LVI, due to factors such as sampling error, cautery artifacts, and interobserver variability, highlight the unmet clinical need for reliable, non-invasive biomarkers [25,31]. Liquid biopsy approaches, including circulating tumor DNA (ctDNA) and non-coding RNA profiles, have shown promising results in the diagnosis of bladder cancer [32,33]. Such biomarkers could be used to identify patients at risk of progression, albeit, to the best of our knowledge, there is no study to evaluate the potential of liquid biopsy in predicting the presence of LVI in such patients. This is particularly important since the literature shows limited concordance between LVI assessment of the TUR specimen vs. that of the cystectomy specimen. Future integration of molecular markers with conventional pathology could enable a more precise and individualized approach to bladder cancer management.

## 6. Study Limitations and Strengths

The main strength of our study lies in its relatively large sample size due to the fact that the hospital is a hub center for the treatment of patients with bladder cancer. This allowed the inclusion of patients from various areas of the country, increasing the generalizability of the results. Moreover, the focus on OS as an outcome provides meaningful clinical insight into prognostic factors in bladder cancer. Despite this, some relevant limitations of the study must be discussed. First, the most important limitation stems from the retrospective design, which might lead to selection bias, incomplete data, and reliance on available medical records or pathology reports. Moreover, the lack of standardized criteria for LVI assessment could influence its prognostic value. Next, subgroup analyses, especially those of Nx patients, had a small sample size, which resulted in borderline significance of the analyses, most likely due to the limited statistical power. We acknowledge the possibility that nodal understaging may have influenced our multivariable analysis. In a perfectly staged cohort, the prognostic contribution of LVI might be partially absorbed by the dominant effect of nodal involvement. Nonetheless, our results underscore the oncological importance of comprehensive lymph node dissection when clinically and surgically feasible.

Another significant limitation is the heterogeneity in peri-operative chemotherapy administration (received by 42% of the cohort). As a retrospective study, these real-world treatment decisions introduce a potential for confounding by indication, as patients selected for chemotherapy likely had different risk profiles. While we accounted for chemotherapy in our models, unmeasured confounders may still influence the observed survival outcomes, and prospective studies are needed to mitigate this bias.

Lastly, OS was used as an endpoint instead of cause-specific mortality, as death data were derived from the national registry, which does not specify the cause of death. Future studies should aim to validate the prognostic significance of LVI in prospective, multicenter cohorts with standardized histopathologic review, as well as in different treatment settings. Combining LVI status with genomic or transcriptomic data may uncover biological mechanisms driving tumor aggressiveness and improve risk stratification.

## 7. Conclusions

Patients with LVI-positive bladder cancer had a significantly worse OS, including those without confirmed LN staging. Given the strong correlation between LVI and nodal involvement, and the established benefit of perioperative systemic therapy in high-risk patients, LVI should be actively integrated into risk-stratification algorithms. Patients with LVI-positive tumors, especially those with limited or no lymph node dissection, should be considered for adjuvant or neoadjuvant chemotherapy and/or immunotherapy rather than managed by surgery alone.

## Figures and Tables

**Table 1:** Demographic, surgical, and pathological characteristics of the patients included in the study.

Variable		LVI-Negative *n* = 122	LVI-Positive *n* = 78	*p*-Value
Age, median (q1; q3)		70 (65; 75)	71 (67; 78)	0.083
Sex, *n* (%)	F	27 (22.13)	13 (16.88)	0.368
	M	95 (77.87)	64 (83.12)	
Smoker, *n* (%)	yes	83 (68.03)	60 (76.92)	0.174
Residential background, *n* (%)	rural	42 (34.43)	29 (37.18)	0.691
	urban	80 (65.57)	49 (62.82)	
Comorbidities, *n* (%)	yes	77 (63.11)	50 (64.1)	0.887
Hypertension, *n* (%)	yes	45 (36.89)	30 (38.46)	0.822
Diabetes mellitus, *n* (%)	yes	34 (27.87)	27 (34.62)	0.312
**Surgery and treatment**				
Time to oncological visit, median (q1; q3)		1 (1; 1)	1 (1; 1)	0.978
Time from diagnosis to cystectomy, median (q1; q3)		3 (2; 5)	2 (1; 4)	0.033
Treatment received, *n* (%)	NAC	41 (33.61)	11 (14.67)	0.001
	AC	11 (9.02)	18 (24)	
	Surgery only	70 (57.38)	46 (61.33)	
Number of NAC cycles, median (q1; q3)		4 (3; 4)	3 (3; 4)	0.103
Postsurgical complications, *n* (%)	yes	11 (9.02)	12 (15.38)	0.169
Preoperative transfusions, *n* (%)	yes	39 (31.97)	32 (41.03)	0.192
Postoperative transfusions, *n*(%)	yes	43 (35.25)	34 (43.59)	0.237
Toxicities, *n* (%)	present	8 (12.31)	2 (5.26)	0.317
CHT dose reduction, *n* (%)	yes	1 (2.22)	1 (5.88)	0.476
**Pathological characteristics**				
Histotype, *n* (%)	urothelial	113 (94.17)	72 (94.74)	1
	clear cell	1 (0.83)	0 (0)	
	squamous	4 (3.33)	3 (3.95)	
	neuroendocrine	2 (1.67)	1 (1.32)	
Grade, *n* (%)	G2	32 (32.99)	6 (8.22)	<0.001
	G3	64 (65.98)	64 (87.67)	
	G4	1 (1.03)	3 (4.11)	
pT, *n* (%)	0	15 (12.4)	0 (0)	<0.001
	1	44 (36.36)	0 (0)	
	2	33 (27.27)	17 (21.79)	
	3	21 (17.36)	31 (39.74)	
	4	8 (6.61)	30 (38.46)	
pT, *n* (%)	≤1	59 (48.76)	0 (0)	<0.001
	≥2	62 (51.24)	78 (100)	
pN, *n* (%)	0	100 (93.46)	33 (50.77)	<0.001
	1	2 (1.87)	16 (24.62)	
	2	4 (3.74)	16 (24.62)	
	3	1 (0.93)	0 (0)	
pN, *n* (%)	negative	100 (93.46)	33 (50.77)	<0.001
	positive	7 (6.54)	32 (49.23)	
Pn, *n* (%)	absent	10 (55.56)	10 (45.45)	0.525
	present	8 (44.44)	12 (54.55)	
Total LN dissected, median (q1; q3)		9 (7; 13)	8 (4; 13)	0.135

Abbreviations: *n*: total number of cases; M: male; F: female; LVI: lymphovascular invasion; NAC: neoadjuvant chemotherapy; AC: adjuvant chemotherapy; CHT: chemotherapy; pT: pathological T stage; pN: pathological N stage; Pn: perineural invasion;. LN: lymph nodes.

**Table 2:** Univariable and multivariable analysis for overall survival and progression-free survival of patients with bladder cancer.

	Univariable Analysis		Multivariable Analysis	
Variable	HR (95%CI)	*p*-Value	HR (95%CI)	*p*-Value
**OS**				
Age (years)	1.03 (1.01; 1.06)	0.009	1.02 (0.99; 1.05)	0.135
Sex (M vs. F)	0.72 (0.46; 1.14)	0.165		
Smoking status (yes vs. no)	0.76 (0.5; 1.15)	0.192		
Comorbidities (present vs. absent)	0.9 (0.6; 1.34)	0.605		
Perioperative chemotherapy (yes vs. no)	0.89 (0.58; 1.36)	0.579		
Post-surgical complications (yes vs. no)	1.35 (0.76; 2.37)	0.304		
Grade (high vs. low)	1.58 (0.93; 2.67)	0.088		
LVI (present vs. absent)	3.13 (2.09; 4.69)	<0.001	1.35 (0.8; 2.26)	0.258
pT (T ≥ 2 vs. T ≤ 1)	6.58 (3.31; 13.1)	<0.001	4.85 (2.19; 10.77)	<0.001
pN (N ≥ 1 vs. N0)	3.42 (2.16; 5.4)	<0.001	1.87 (1.13; 3.09)	0.015
**PFS**				
Age (years)	1.03 (1; 1.06)	0.056		
Sex (M vs. F)	0.69 (0.39; 1.22)	0.203		
Smoking status (yes vs. no)	0.82 (0.5; 1.36)	0.447		
Comorbidities (present vs. absent)	0.54 (0.33; 0.91)	0.019	0.74 (0.42; 1.3)	0.299
Perioperative chemotherapy (yes vs. no)	0.56 (0.33; 0.94)	0.028	0.5 (0.29; 0.87)	0.015
Post-surgical complications (yes vs. no)	0.95 (0.38; 2.37)	0.907		
Grade (high vs. low)	1.98 (0.85; 4.63)	0.114		
LVI (present vs. absent)	2.57 (1.53; 4.3)	<0.001	1.47 (0.84; 2.56)	0.180
pT (T ≥ 2 vs. T ≤ 1)	6.57 (2.61; 16.54)	<0.001	5.52 (2.04; 14.93)	0.001
pN (N ≥ 1 vs. N0)	1.09 (1.01; 1.17)	0.024	1.03 (0.95; 1.12)	0.484

Abbreviations: LVI: lymphovascular invasion; M: male; F: female; pT: pathological T stage; pN: pathological N stage.

**Table 3:** Univariable and multivariable analysis for overall survival and progression-free survival of patients with bladder cancer undergoing cystectomy without systemic chemotherapy.

	Univariable Analysis		Multivariable Analysis	
Variable	HR (95%CI)	*p*-Value	HR (95%CI)	*p*-Value
**OS**				
Age (years)	1.03 (1; 1.06)	0.023	1.02 (0.99; 1.06)	0.160
Sex (M vs. F)	1.13 (0.57; 2.21)	0.729		
Smoking status (yes vs. no)	0.85 (0.50; 1.45)	0.552		
Comorbidities (present vs. absent)	1.08 (0.66; 1.75)	0.760		
Post-surgical complications (yes vs. no)	1.05 (0.48; 2.3)	0.902		
Grade (high vs. low)	1.39 (0.75; 2.56)	0.292		
LVI (present vs. absent)	4 (2.41; 6.66)	<0.001	1.66 (0.84; 3.30)	0.143
pT (T ≥ 2 vs. T ≤ 1)	6.70 (3.04; 14.73)	<0.001	4.81 (1.85; 12.49)	0.001
pN (N ≥ 1 vs. N0)	4.17 (2.35; 7.39)	<0.001	1.92 (1.02; 3.62)	0.015
**PFS**				
Age (years)	1.01 (0.97; 1.04)	0.629		
Sex (M vs. F)	0.89 (0.33; 2.35)	0.809		
Smoking status (yes vs. no)	1.33 (0.64; 2.78)	0.448		
Post-surgical complications (yes vs. no)	0.32 (0.04; 2.33)	0.259		
Grade (high vs. low)	1.17 (0.35; 3.96)	0.796		
LVI (present vs. absent)	4.32 (1.89; 9.86)	0.001	2.19 (0.91; 5.24)	0.078
pT (T ≥ 2 vs. T ≤ 1)	12.42 (2.91; 52.89)	0.001	7.18 (1.49; 34.56)	0.014
pN (N ≥ 1 vs. N0)	1.11 (1; 1.22)	0.042	1.03 (0.92; 1.15)	0.633

Abbreviations: LVI: lymphovascular invasion; M: male; F: female; pT: pathological T stage; pN: pathological N stage.

**Table 4:** Univariable and multivariable analysis for overall survival of patients with bladder cancer and negative lymph node status.

	Univariable Analysis		Multivariable Analysis	
Variable	HR (95%CI)	*p*-Value	HR (95%CI)	*p*-Value
**OS**				
Age (years)	1.03 (1; 1.06)	0.036	1.03 (1.01; 1.06)	0.040
Sex (M vs. F)	0.60 (0.35; 1.02)	0.060		
Smoking status (yes vs. no)	0.64 (0.39; 1.05)	0.077		
Comorbidities (present vs. absent)	0.80 (0.5; 1.29)	0.364		
Perioperative chemotherapy (yes vs. no)	1.11 (0.61; 2.02)	0.724		
Post-surgical complications (yes vs. no)	1.04 (0.5; 2.18)	0.910		
Grade (high vs. low)	1.20 (0.68; 2.11)	0.522		
LVI (present vs. absent)	2.71 (1.68; 4.37)	<0.001	1.41 (0.85; 2.35)	0.179
pT (T ≥ 2 vs. T ≤ 1)	5.44 (2.7; 11)	<0.001	4.69 (2.22; 9.91)	0.001
**PFS**				
Age (years)	1.03 (0.99; 1.07)	0.129		
Sex (M vs. F)	0.97 (0.39; 2.37)	0.939		
Smoking status (yes vs. no)	1.05 (0.51; 2.13)	0.903		
Comorbidities (present vs. absent)	0.83 (0.38; 1.81)	0.645		
Perioperative chemotherapy (yes vs. no)	0.71 (0.34; 1.5)	0.372		
Post-surgical complications (yes vs. no)	0.69 (0.16; 2.89)	0.608		
Grade (high vs. low)	1.76 (0.61; 5.08)	0.294		
LVI (present vs. absent)	3.01 (1.46; 6.22)	0.003	1.69 (0.78; 3.67)	0.186
pT (T ≥ 2 vs. T ≤ 1)	4.87 (1.86; 12.77)	0.001	3.89 (1.38; 10.98)	0.010

Abbreviations: LVI: lymphovascular invasion; M: male; F: female; pT: pathological T stage.

**Table 5:** Univariable analysis for overall survival and progression-free survival of patients with bladder cancer undergoing cystectomy with Nx status.

	Univariable Analysis	
Variable	HR (95%CI)	*p*-Value
**OS**		
Age (years)	1.02 (0.96; 1.08)	0.579
Sex (M vs. F)	0.89 (0.34; 2.34)	0.806
Smoking status (yes vs. no)	0.4 (0.14; 1.15)	0.090
Comorbidities (present vs. absent)	1.01 (0.4; 2.5)	0.991
Perioperative chemotherapy (yes vs. no)	0.87 (0.33; 2.3)	0.774
Post-surgical complications (yes vs. no)	1.39 (0.31; 6.2)	0.667
Grade (high vs. low)	1.26 (0.36; 4.36)	0.718
LVI (present vs. absent)	2.23 (0.88; 5.66)	0.091
**PFS**		
Age (years)	0.94 (0.83; 1.07)	0.342
Sex (M vs. F)	0.52 (0.14; 1.95)	0.333
Smoking status (yes vs. no)	0.7 (0.2; 2.43)	0.574
Comorbidities (present vs. absent)	0.67 (0.18; 2.5)	0.553
Perioperative chemotherapy (yes vs. no)	0.35 (0.09; 1.34)	0.125
Post-surgical complications (yes vs. no)	0.55 (0.11; 2.77)	0.472
Grade (high vs. low)	1.81 (0.52; 6.32)	0.354
LVI (present vs. absent)	0.94 (0.83; 1.07)	0.342

Abbreviations: LVI: lymphovascular invasion; M: male; F: female; pT: pathological T stage.

## Data Availability

The original contributions presented in this study are included in the article/Supplementary Material. Further inquiries can be directed to the corresponding author.

## Supplementary Materials

The following supporting information can be downloaded at: https://www.mdpi.com/article/10.3390/jcm14145120/s1, Figure S1: STROBE flowchart showing the selection process of the patients included in the study. Table S1: Verification of non-proportional hazards of the COX analyses on bladder cancer patients. Figure S2: Kaplan-Meier curves showing the overall survival of patients divided based on their LVI status and treatment type. Figure S3: Kaplan-Meier curves showing the overall survival of patients with N+ lymph nodes divided based on their LVI status.
