# Supplementary material for: Lymphovascular Invasion Is a Predictor of Clinical Outcomes in Bladder Cancer Patients Treated with Radical Cystectomy
Source: J Clin Med. 2025 Jul 18;14(14):5120. doi: 10.3390/jcm14145120 (PMC12295869; doi:10.3390/jcm14145120)
Supplement: Supplementary file 1 [file jcm-14-05120-s001.zip › jcm-3698627-supplementary.pdf]

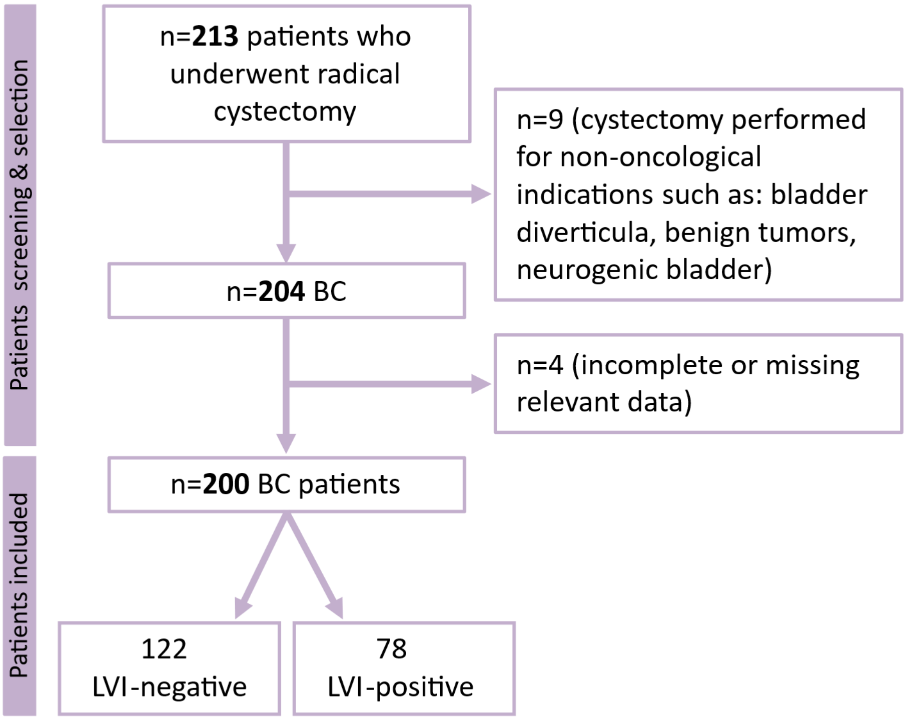

Supplementary Figure S1. STROBE flowchart showing the selection process of the patients included in the study.

Supplementary Table S1. Verification of non-proportional hazards of the COX analyses on bladder cancer patients.

	OS			PFS		
**Variable**	**chisq**	**df**	**p-value**	**chisq**	**df**	**p-value**
Age (years)	0.02	1	0.877	0.78	1	0.38
Sex (M vs F)	0.27	1	0.603	2.22	1	0.14
Smoking status (yes vs no)	1.37	1	0.242	0.01	1	0.92
Comorbidities (present vs absent)	0.37	1	0.542	0.01	1	0.94
Perioperative chemotherapy (yes vs no)	2.51	1	0.113	0.00	1	0.99
Post-surgical complications (yes vs no)	0.21	1	0.644	0.07	1	0.79
Grade (high vs low)	0.01	1	0.928	0.00	1	0.99
LVI (present vs absent)	0.33	1	0.568	0.17	1	0.68
pT (T≥2 vs T≤1)	0.11	1	0.739	0.74	1	0.39
pN (N≥1 vs N0)	3.70	1	0.054	0.00	1	0.96
Global	13.51	10	0.197	5.07	10	0.89

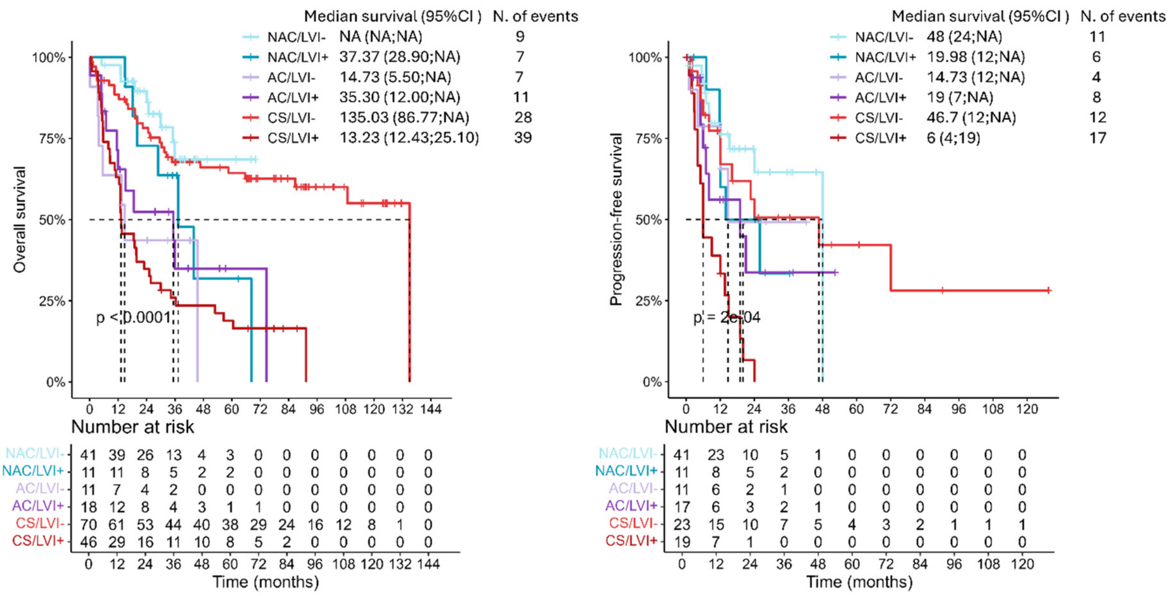

Supplementary Figure S2. Kaplan-Meier curves showing the overall survival of patients divided based on their LVI status and treatment type. *LVI: lymphovascular invasion, NAC: neoadjuvant therapy, AC: adjuvant therapy, CS: cystectomy only (without any chemotherapy).*

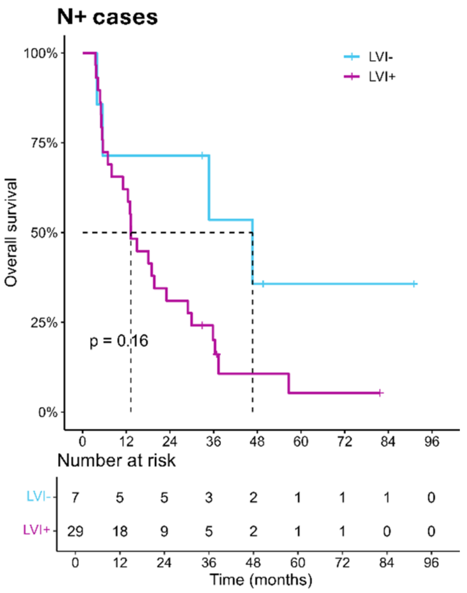

Supplementary Figure S3. Kaplan-Meier curves showing the overall survival of patients with N+ lymph nodes divided based on their LVI status. *LVI: lymphovascular invasion.*
